# The effects of paternal obesity on sperm characteristics and early embryonic development: results from a retrospective study

**DOI:** 10.1186/2049-3258-73-S1-P47

**Published:** 2015-09-17

**Authors:** Tessy Boedt, Koen De Winne, Carl Spiessens, Adelheid Soubry

**Affiliations:** 1 KU Leuven, Leuven, Belgium; 2 UZ Leuven, Leuven, Belgium

## 

In humans, it is widely demonstrated that maternal obesity negatively affects fertility, pregnancy outcomes and offspring's health. Less is known about the effects of paternal obesity on fertility and offspring's development. Recent observations in animal models show that diet-induced obese males have impaired sperm quality, delayed preimplantation growth of the fertilized oocyte, and reduced implantation rate. Our aim was to evaluate the effects of paternal obesity on sperm quality and subsequently embryonic development and implantation in humans. Body mass index (BMI), sperm and embryo parameters of 192 men who participated in a fertility program (IVF or ICSI) at the Leuven University Fertility centre between March and September 2014 were evaluated. Sperm parameters included: concentration, total sperm count, motility and morphology. Embryo parameters included number and asymmetry of blastomeres, degree of fragmentation and implantation success. The embryonic development parameters were obtained using a new computer assisted scoring system (CASS) based on multilevel image analyses. Our analytic study included sperm data from 192 men and embryo characteristics from 138 couples. Multiple regression models were used to determine potential associations between paternal obesity/BMI and sperm or embryo parameters. No significant associations were found between paternal obesity/BMI and the sperm parameters measured. After adjusting for potential confounding, we found a borderline significant effect of paternal obesity/BMI on the degree of asymmetry of blastomeres: coefficient was +0.05 (p=0.047) if men were obese, and coefficient was +0.004 (p=0.059) with increasing paternal BMI. This effect was only seen on day 2 of embryonic development. No effect of paternal obesity/BMI on implantation success was found. In order to confirm a potential effect of obesity on embryonic development our analyses will be further extended on a larger dataset. We further aim to gain insights on the underlying epigenetic mechanisms that have been reported as being mediated by paternal obesity.

